# Phase I/II clinical trial of the targeted chemotherapeutic drug, folate-tubulysin, in dogs with naturally-occurring invasive urothelial carcinoma

**DOI:** 10.18632/oncotarget.26455

**Published:** 2018-12-11

**Authors:** Nicholas M. Szigetvari, Deepika Dhawan, José A. Ramos-Vara, Christopher P. Leamon, Patrick J. Klein, A. Audrey Ruple, Hock Gan Heng, Michael R. Pugh, Satish Rao, Iontcho R. Vlahov, Pierre L. Deshuillers, Philip S. Low, Lindsey M. Fourez, Ashleigh M. Cournoyer, Deborah W. Knapp

**Affiliations:** ^1^ Department of Veterinary Clinical Sciences, Purdue University, West Lafayette, IN, USA; 2 Department of Comparative Pathobiology, Purdue University, West Lafayette, IN, USA; 3 Endocyte Inc., West Lafayette, IN, USA; ^4^ Department of Chemistry, Purdue University, West Lafayette, IN, USA; ^5^ Purdue University Center for Cancer Research, West Lafayette, IN, USA

**Keywords:** animal model, bladder cancer, canine, clinical trial, targeted chemotherapy

## Abstract

**Purpose:**

The purpose was to determine the safety and antitumor activity of a folate-tubulysin conjugate (EC0531) in a relevant preclinical animal model, dogs with naturally-occurring invasive urothelial carcinoma (iUC). Canine iUC is an aggressive cancer with high folate receptor (FR) expression similar to that in certain forms of human cancer.

**Experimental Design:**

A 3+3 dose escalation study of EC0531 (starting dose 0.2 mg/kg given intravenously at two-week intervals) was performed in dogs with iUC expressing high levels of FRs (>50% positive tumor cells). Pharmacokinetic (PK) analysis was performed, and the maximum tolerated dose (MTD) was determined. The dose cohort at the MTD was expanded to determine antitumor activity.

**Results:**

The MTD of EC0531 was 0.26 mg/kg every two weeks, with grade 3-4 neutropenia and gastrointestinal toxicity observed at higher doses. Treatment at the MTD was well tolerated. Clinical benefit was found in 20 of 28 dogs (71%), including three dogs with partial remission and 17 dogs with stable disease. Plasma EC0531 concentrations in the dogs far exceeded those required to inhibit proliferation of FR-expressing cell *in vitro*. Unlike human neutrophils, canine neutrophils were found to express FRs, which contributes to the neutropenia at higher doses of EC0531 in dogs.

**Conclusion:**

EC0531 was well tolerated and had good antitumor activity in dogs with iUC. It is likely that humans will tolerate higher, potentially more effective doses of folate-tubulysin without myelotoxicity because of the absence of FRs on human neutrophils. The results clearly justify the evaluation of folate-tubulysin in human clinical trials.

## INTRODUCTION

Targeted chemotherapy allows the selective delivery of a cytotoxic payload to tumor cells while limiting the exposure and toxicity to normal tissues. The resulting preferential accumulation of drug in the tumor can increase drug activity with lower systemic exposure, i.e. increasing the therapeutic index. In this way, targeted delivery of cytotoxic drugs allows reconsideration of compounds previously deemed clinically irrelevant due to very low therapeutic index.

One promising, emerging, druggable target for cancer therapy is folate receptor alpha (FRα), which is a high-affinity receptor for folate (vitamin B9) [1, 2]. Folate is needed by all cells for DNA synthesis [1, 2]. Most mammalian cells utilize reduced folate (5-methyl-tetrahydrofolate), which is transported intracellularly via the reduced folate carrier [1–3]. In contrast, non-reduced folate is utilized by tumor cells, and typically transported via high-affinity folate receptors (FRs), mainly FRα in solid tumors [4]. Several human cancer types, such as ovarian carcinoma, renal carcinoma, non-small cell lung cancer, colorectal cancer, and metastatic brain cancer, have 10- to 100-fold higher expression of FRs relative to normal tissues [2, 4]. The high expression of FRs can be exploited for the delivery of cytotoxic drugs conjugated to folate [2–5]. Thus, folate-targeted drugs are expected to largely spare normal tissues by preferentially binding to tumor cells of select cancer types.

Several conjugates of folate and cytotoxic drugs or folate and immune-modulating drugs have been developed in an attempt to exploit this disparity in FR expression [2, 3, 6]. One of the more extensively studied is a desacetylvinblastine monohydrazide (DAVLBH) folate conjugate (vintafolide, EC145) [2, 3, 5]. In a tumor xenograft mouse model, administration of free DAVLBH created unacceptable toxicity before effective anti-tumor activity could be observed [7]. Yet, conjugation of the drug to folate was well tolerated, and led to complete remission of multi-drug resistant tumors in mice [2, 3, 7]. Subsequently, a phase I trial demonstrated an acceptable safety profile of vintafolide in people receiving this drug for an assortment of solid tumors [8]. Varying degrees of clinical effectiveness were observed in phase II and III trials, with the greatest benefit noted in patients with tumors with the highest FR expression and tumors lacking P-glycoprotein expression [2, 3, 5, 9]. These findings were encouraging for future targeting of FRs, but also indicated the need for additional, potentially more active drug conjugates.

Tubulysins A, B, C, and D are potent anti-mitotic compounds isolated from a mycobacterium, *Archangium gephyra*, found in a German soil sample over 40 years ago [10]. The mechanism of action is similar to that of the vinca alkaloids and dolastatins, causing instability of microtubule formation and subsequent cell cycle arrest in late G2/M phase [10]. However, tubulysins have several potential advantages over other anti-mitotic compounds. Unlike vinca alkaloids, tubulysins do not appear to be a substrate for CYP metabolism, thus there is less concern for drug-to-drug interactions [11]. Tubulysins are also poor substrates for the P-glycoprotein drug efflux pump, thus retaining efficacy against multi-drug resistant (MDR) tumor phenotypes [11]. *In vitro*, tubulysin A had superior potency against colon cancer cells with high P-glycoprotein expression (HCT-15 cells), compared to drugs that are P-glycoprotein substrates [11]. The concentration of tubulysin A needed to inhibit cell proliferation by 50% (IC50) of HCT-15 cells was 200 and 2,000 times less than the IC50 of vinblastine and paclitaxel, respectively [11]. Furthermore, growth inhibition was observed in 33% of cell lines tested in a standardized cancer cell line panel, the U.S. National Cancer Institute 60 tumor cell lines for anticancer drug screen. These results are better than those for 95% of the other compounds tested in the same manner by the National Cancer Institute [11]. Despite this compelling information, the clinical effectiveness of tubulysins has not been well established due to the very low therapeutic index.

Administration of free tubulysin creates unacceptable toxicity in mice, even at sub-therapeutic dosages [12]. Yet, conjugation of tubulysin, specifically tubulysin B, to folate creates a drug tolerated at doses 20 times higher than doses of unconjugated tubulysin, and a drug which has induced complete regression of cancer in mouse xenograft models [12–14]. The insertion of a specific molecular spacer, 1-amino-1-deoxy-glucitolyl-γ-glutamate, in folate-tubulysin conjugates was especially effective in increasing the therapeutic range of the resulting compound (EC0531, Endocyte, Inc., West Lafayette, IN) [14]. There is considerable interest in further preclinical (pre-human) evaluation of folate-tubulysin conjugates, especially in animal cancer models that are relevant to human cancer in regards to tumor heterogeneity, metastatic potential, tumor-host immune interactions, and inherent or acquired drug resistance.

Dogs with specific forms of naturally-occurring cancer, can serve as these highly relevant, *in vivo* models of human cancer in which the outcome of new compounds in dogs is expected to mimic that in humans [15–21]. There are multiple advantages of studying pet dogs with specific forms of naturally-occurring cancer as models for those same cancers in humans. These similarities include tumor histopathology, heterogeneity, genetic and epigenetic alterations, biological behavior, frequent distant metastases, and response to therapy [17–24]. Likewise, similarities in FR expression have been reported between dogs and humans [16, 24]. Normal canine tissues have relatively low FR expression compared to certain canine tumor types, just as normal human tissues have relatively low FR expression compared to tumor tissues [24]. In one of the more extensively studied forms of canine cancer, invasive urothelial carcinoma (iUC), FR over-expression was noted in 76% of iUC cases, and folate uptake in the cancer was confirmed by scintigraphy [24]. In this same study, 78% of human iUC cases had increased expression of FRs when detected using the same antibody as in the canine tissues, although the expression level was lower when detected by other antibodies [24]. Canine FRα protein has >80% predicted homology with the human FRα.

The goal of the study reported here was to determine the safety and antitumor activity of a folate-tubulysin B drug conjugate (EC0531) in a canine spontaneous cancer model that incorporates factors which would affect drug response in humans such as tumor heterogeneity, metastatic behavior, immune system interactions, and innate and acquired drug resistance. Specifically, the objectives of the study were to: (1) determine the safety, tolerability, maximum tolerated dose (MTD), and pharmacokinetics of EC0531 in pet dogs with naturally-occurring urinary tract iUC, and (2) determine antitumor activity in the dogs with spontaneously arising iUC with high FR expression.

## RESULTS

### Case enrollment and establishing the MTD of EC0531

**Figure 1:**
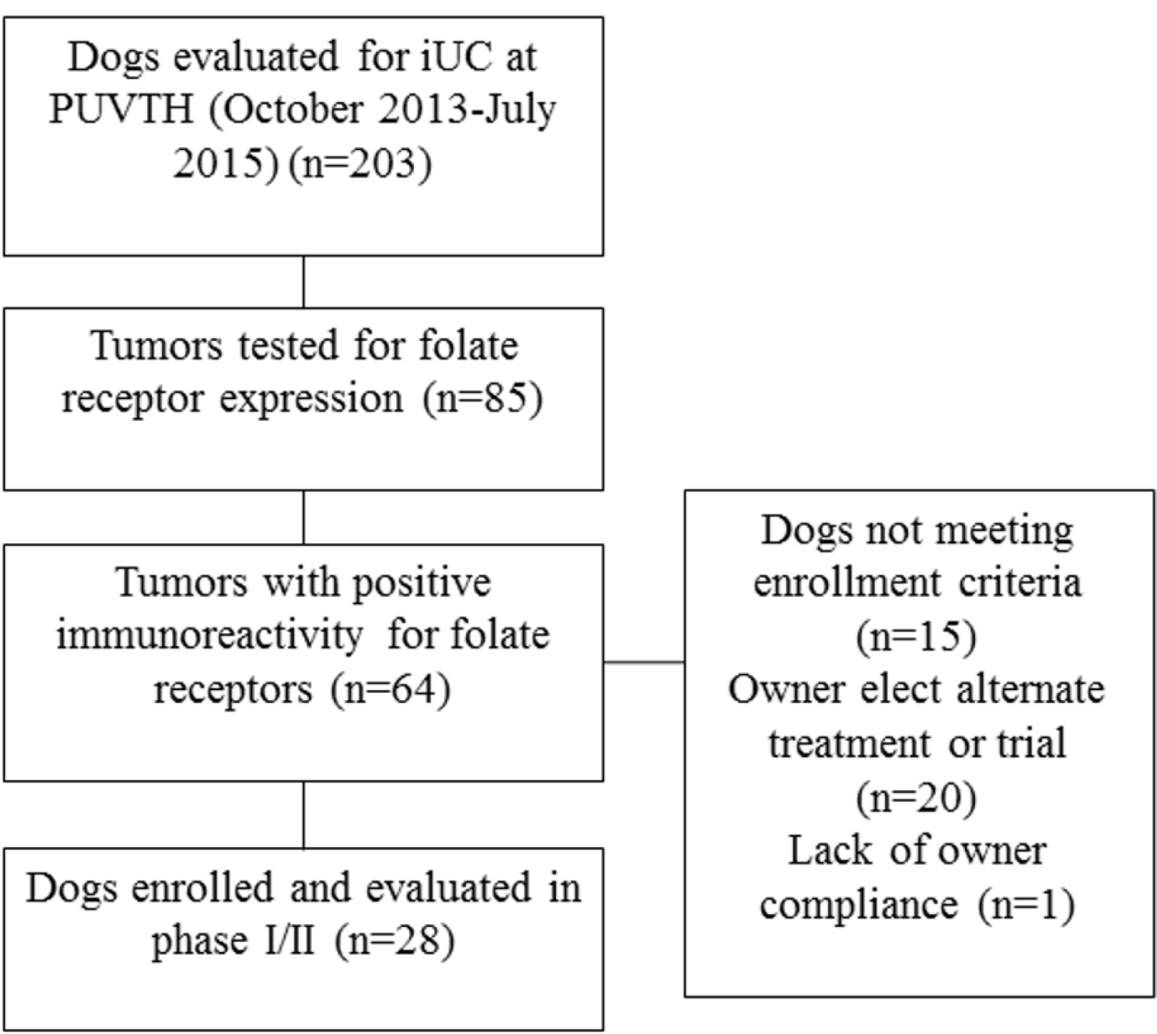
Summary of screening and enrollment of dogs in the EC0531 trial

The selection of dogs for the phase I/II trial is summarized in Figure 1. Fifteen dogs were included in the work to establish the MTD of EC0531. The AEs are summarized in Table 1. The cohort was expanded when a DLT was observed in the cohort of dogs receiving 0.28 mg/kg EC0531. The cohort included four dogs that had initially received a lower EC0531 dose and had undergone intra-patient dose escalation to receive 0.28 mg/kg, and six additional dogs who received 0.28 mg/kg as their initial dose of EC0531. The AEs observed in dogs receiving 0.28 mg/kg exceeded those allowed for the MTD. Six dogs were treated with the next lower dose (0.26 mg/kg), and this dose was established as the MTD. There was variation in the drug tolerability from dog to dog with some dogs tolerating escalated doses above the MTD (Table 1). For example, one dog tolerated EC0531 doses of 0.30 and 0.32, and initial doses of 0.34 mg/kg before developing G3 anorexia after the fifth dose of 0.34 mg/kg.

**Table 1:** Adverse events occurring in dogs in the phase I escalation study of EC0531

Dose of EC0531	Number of dogs receiving that dose	Number of doses given	Adverse events defined by VCOG-CTCAE (G–Grade; DLT–Dose limiting toxicity)
0.18	1	6	1 dog with G1 anorexia
0.20	4	14	1 dog with G2 anorexia
0.22	4	10	1 dog with G1 lethargy
0.24	4	16	1 dog with G2 neutropenia
0.26	6	33	1 dog with G1 lethargy 1 dog with G1 vomiting and G1 neutropenia 1 dog with G2 neutropenia 1 dog with G3 neutropenia (DLT) and G2 diarrhea
0.28	10^a,b^	63	1 dog with G1 vomiting^a^ 1 dog with G1lethargy and G3 weight loss (DLT)^a^ 1 dog with G4 neutropenia (DLT)^b^ 1 dog with G4 infusion reaction (DLT)^b^
0.30	2	15	1 dog with G3 neutropenia (DLT)
0.32	1	2	No adverse events observed
0.34	1	5	1 dog with G3 anorexia (DLT)

^a^Four dogs initially received a lower dose of EC0531, and then underwent intra-patient dose escalation to receive 0.28 mg/kg EC0531.

^b^Six new dogs were enrolled for which their initial dose of EC0531 was 0.28 mg/kg.

An additional 13 dogs, or 28 dogs total, were enrolled in the phase I/II trial. The expression of FRs was detected by IHC on biopsy samples in 25 dogs and by ICC on tumor cells in urine sediment in three dogs. The dogs had a median age of 11.4 years (7.1–16.5 years) and median weight of 17.5 kg (2.5–33.0 kg), and included 21 spayed female and seven neutered male dogs. The most common breed represented was mixed breed (*n* = 12), followed by Scottish Terrier (*n* = 4), Dachshund (*n* = 2), Yorkshire Terrier (*n* = 2), and one of each of the following: Norwegian Elkhound, Irish Setter, American Eskimo Dog, Fox Terrier, Border Collie, West Highland White Terrier, Boxer, and Tibetan Terrier.

The primary tumor was present in the bladder in 10 dogs, the urethra in two dogs, and the bladder plus the urethra/prostate in 16 dogs. Two dogs had distant metastases, and one dog had nodal metastases at diagnosis. Twelve dogs had failed prior therapy with a cyclooxygenase inhibitor (COXi) and continued receiving COXi therapy during the trial. Five dogs had failed prior chemotherapy including, vinblastine, carboplatin, mitoxantrone, and toceranib.

The 28 dogs received a total of 311 doses of EC0531, and the drug was generally well tolerated. Intra-patient dose escalation permitted expansion of the cohort of dogs receiving EC0531 at the MTD. Twenty-six of the 28 dogs in this study received EC0531 at or above the MTD. Toxicity noted consisted of neutropenia, anorexia, or lethargy. Eosinopenia accompanied the neutropenia. One dog experienced grade 2 thrombocytopenia. This dog had been heavily pretreated with chemotherapy prior to enrolling in the EC0531 trial, and had chronically low platelet counts. The platelet count was lower at a week after EC0531 treatment, and then rebounded to pre-EC0531 levels by day 14. Several dogs with late stage cancer or urethral/ureteral obstruction developed azotemia and weight loss during the end of their treatment as the cancer was beginning to progress. These findings, however, were still recorded as AEs. These were the only AEs that were not transient. No dog withdrew from the study due to AE or negative effects towards quality of life. A single dog developed an infusion reaction consisting of hypotension, compensatory tachycardia, and subsequent syncopal episode. The reaction was transient and resolved within three minutes without intervention. The dog had failed multiple other therapies, and achieved remission with EC0531 treatment, and therefore, the EC0531 treatment was continued. Slowing the rate of administration did not alleviate the reaction. The administration of diphenhydramine partially alleviated the clinical signs. This dog continued to benefit from treatment, and received 11 doses of EC0531.

### Antitumor activity of EC0531

**Figure 2:**
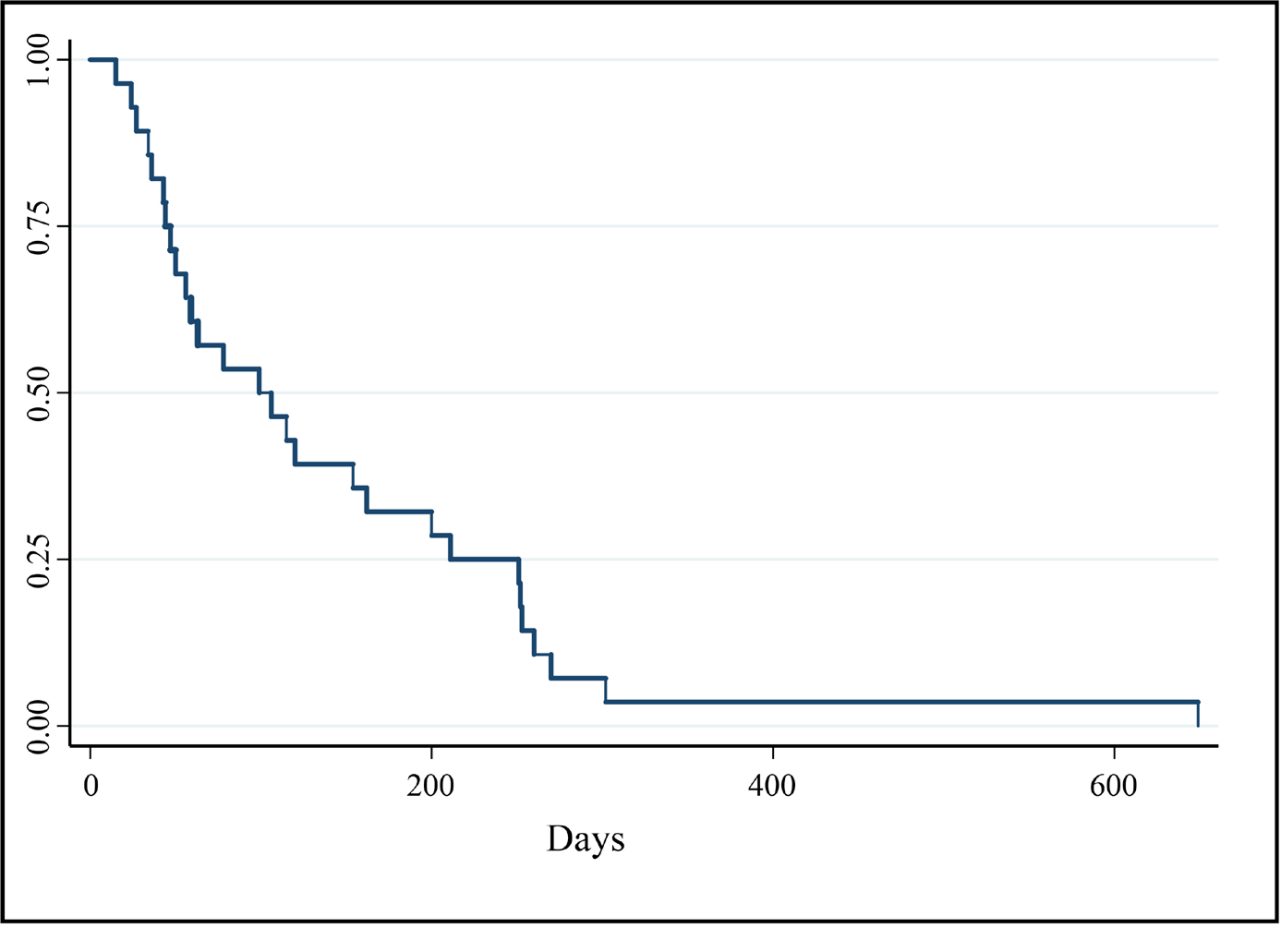
Kaplan–Meier survival curve of PFI (days) for all dogs enrolled in phase I and II portions of clinical trial (*n* = 28) Median PFI = 103 days.

Clinical benefit was found in 20 of the 28 dogs (71%), including PR in three dogs and SD in 17 dogs. The median PFI for all dogs was 103 days (range 24–649 days) (Figure 2). The two dogs receiving all of their doses of EC0531 below the MTD had SD and PD, respectively, of their tumors. Nineteen dogs had PD recorded at time of last follow-up. Cancer progression occurred at the primary site in 12 dogs, metastatic sites in two dogs, and both the primary and metastatic sites in five dogs. The two variables found to have significant effects on PFI were best tumor response and receiving prior treatment with chemotherapy (*p* = 0.0001). Non-responders, i.e. dogs with PD after the initial two treatments, had a median PFI of 34 days (range 24–47). Dogs with SD had a median PFI of 200 days (range 56–296 days). The PFI or time of censoring of the three dogs achieving PR was 162, 251, and 649 days, respectively. The PFI was significantly different between dogs with SD and those with PD [hazard ratio = 0.013 (95% CI 0.001–0.124)], and was different between dogs achieving PR and dogs with PD [hazard ratio = 0.003 (95% CI 0–0.066)]. Dogs that had received and failed prior chemotherapy had a median PFI of 95 days (range 34–162) with HR of 4.6 (95% CI 1.2–16.7) for earlier PD. There was no association between the level of FR expression in the pre-treatment biopsy and the tumor response or PFI.

Nine dogs were censored from PFI data. One dog was censored at the time it began receiving a COXi to control severe, unrelated, osteoarthritis pain. COXi medications have been noted to have antitumor activity against canine iUC [16]. One dog had two additional tumor types found at necropsy that were suspected to have resulted in the decline in quality of life leading to euthanasia. Four dogs were euthanized because of owner concern for poor quality of life due to persistent clinical signs, but in the absence of tumor progression. Lastly, in three dogs in which the cancer was still controlled, the dog owners withdrew the dogs from the trial in an attempt to achieve cancer remission with other therapies. All three of those dogs had achieved SD on EC0531.

### Pharmacokinetic analysis

**Figure 3:**
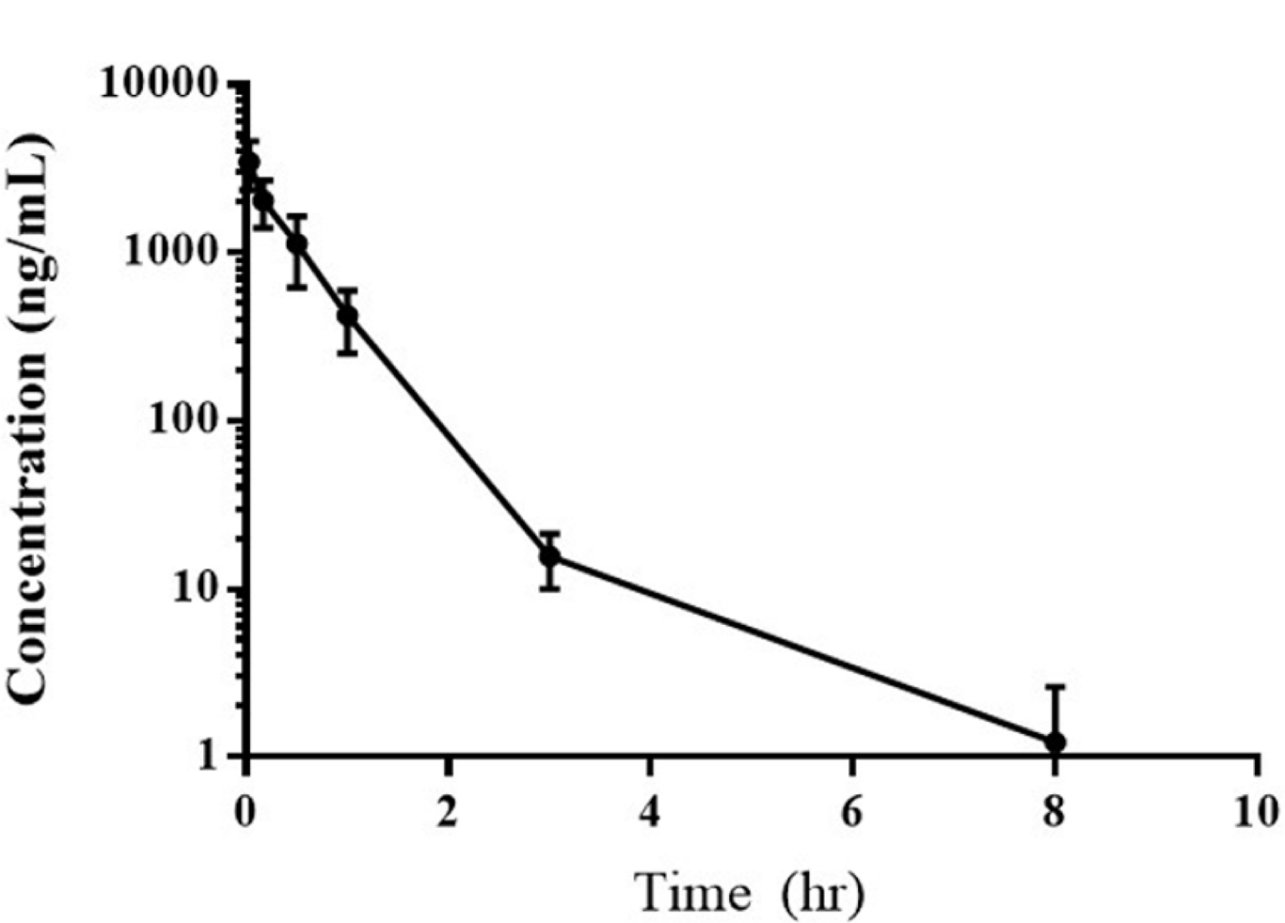
Average plasma concentration of EC0531 versus time following the intravenous administration of 0.28 mg/kg of the drug

The results of the pharmacokinetic analysis of samples from six dogs receiving 0.28 mg/kg EC0531 are summarized in Table 2 and Figure 3. As expected, EC0531 exhibited biphasic pharmacokinetic behavior, and was rapidly cleared from circulation with an elimination half-life of approximately 25 minutes. Importantly, the pharmacokinetic parameters in the dog that had the infusion reaction (dog #3 in Table 2) were not different from those in the other dogs. The concentration of EC0347 (released, active parent tubulysin compound) in the plasma of this dog was minimal (3.2 ng/mL) and was similar to the EC0347 concentration (4.0 ng/mL) in another dog treated at the same time (dog #2 in Table 2).

**Table 2:** Pharmacokinetic parameters from six dogs treated with a single EC0531 dose of 0.28 mg/kg given intravenously

Dog #	t_1/2_ (min)	T_max_ (min)	C_max_ (ng/mL)	C_0_ (ng/mL)	AUC_last_ (hr*ng/mL)	AUC_inf_ (hr*ng/mL)	V_z_ (L/kg)	Cl (L/hr/kg)	T_max_ (min)^	C_max_ (ng/mL)^
1	22.0	2.0	4400	4971	2299	2305	0.0643	0.121	10.0	2.0
2	19.9	2.0	5270	6187	2801	2806	0.0477	0.100	10.0	4.0
3	26.3	2.0	2630	2840	1552	1563	0.1134	0.179	10.0	3.2
4	26.5	2.0	2920	3335	1679	1681	0.1062	0.167	10.0	2.2
5	26.4	2.0	3300	3641	2082	2084	0.0853	0.134	2.0	1.8
6	27.3	2.0	2410	2976	939	941	0.1956	0.298	30.0	0.7
Avg	24.7	2.0	3488	3992	1892	1896	0.102	0.167	12.0	2.3
SD	3.03	0	1119	1319	647	648	0.052	0.071	9.4	1.1

The dogs included (in order in the table): a 15 year old, 23 kg, neutered male Norwegian Elkhound; a 9 year old, 27 kg, spayed female mixed breed dog; a 13 year old, 7 kg, spayed female Tibetan Terrier; a 14 year old, 13 kg, neutered male Fox Terrier; an 11 year old, 17 kg, spayed female Border Collie; and a 9 year old, 11 kg, spayed female Scottish Terrier.

^^^EC0347 (free drug) data.

The concentrations of EC0531 and EC0347 were measured in iUC tissues in three dogs at approximately three hours after treatment. The concentration of EC0531 was below the level of detection in two dog samples, and was 3.9 ng/mL in the sample from the third dog. The EC0347 concentrations were 5.5, 7.3, and 13.8 ng/mL, respectively, in the tissues from these three cases. All three dogs had received 0.28 mg/kg EC0531, and all three dogs had a tumor response of SD.

### FR expression on neutrophils and neutrophil precursors

**Figure 4:**
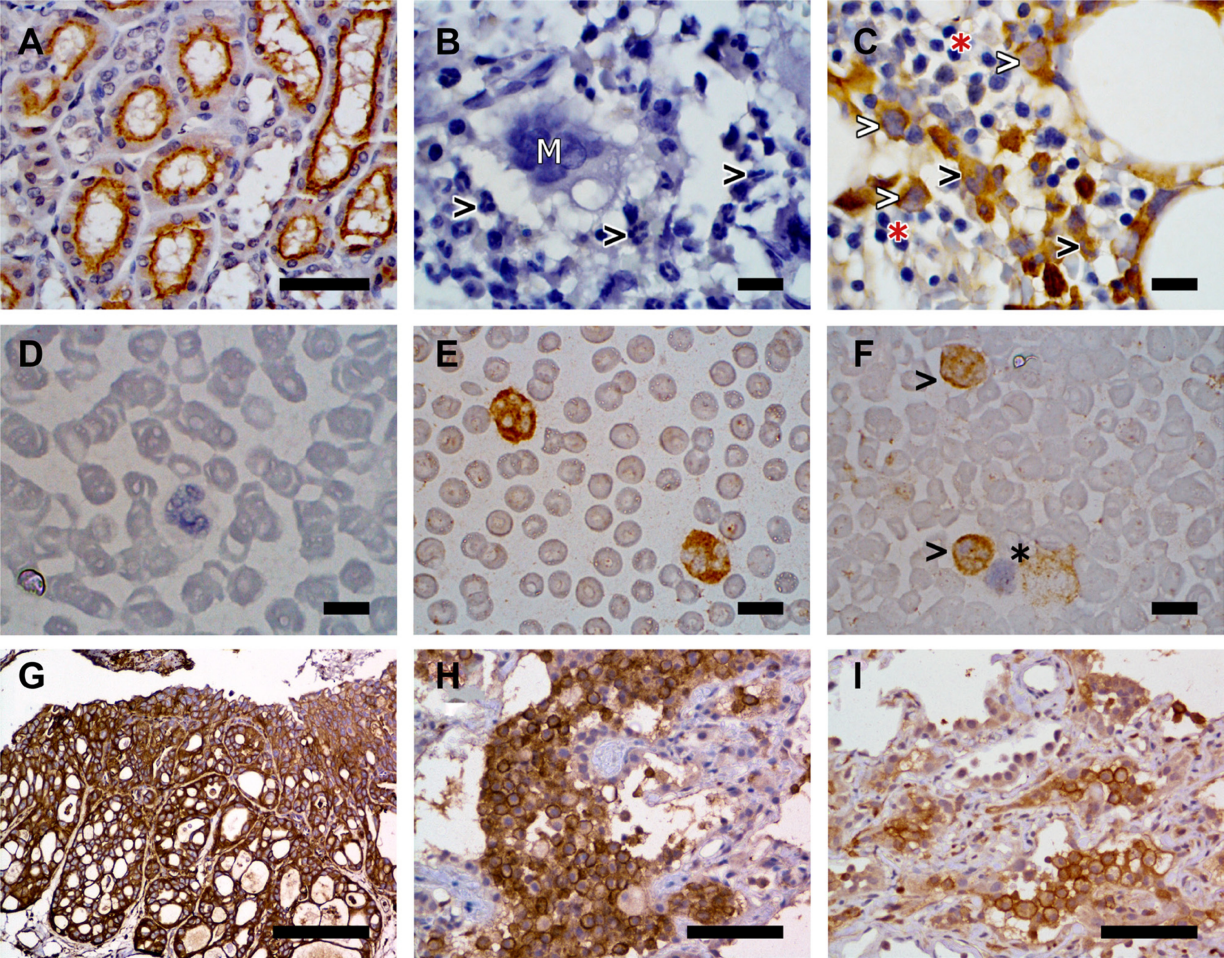
Representative examples of FR expression detected on canine kidney (**A**), canine bone marrow sections (**B, C**), canine peripheral blood smears (**D, E, F**), and canine iUC (**G, H, I**). FR expression was detected by immunohistochemistry in panels (A–C) and (G–H), and by immunocytochemistry in panels (D–F). (A) Canine kidney used as a positive control. Immunoreactivity is detected on the apical surface of the proximal renal tubular epithelium. Image taken at 40× magnification. Scale bar = 50 µm. (B) Canine bone marrow section used as negative control. Note the absence of immunoreactivity in granulocytes (black arrowheads) and megakaryocytes (white “M”). (C) Paired bone marrow sample showing FR expression by early myeloid precursor cells (white arrowheads) and granulocytes (black arrowhead). Erythroid precursors (red asterisk) do not display immunoreactivity. (D) Canine peripheral blood smear negative control. (E) Paired specimen showing marked cytoplasmic and membranous immunoreactivity in granulocytes. (F) Absence of FR immunoreactivity on lymphocytes (black asterisk) contrary to positive immunoreactivity on granulocytes (black arrowheads). Images B to F were taken at 100× magnification. Scale bars = 10 µm. (G) Canine iUC, urinary bladder. Strong labeling of neoplastic cells for FR is observed. Scale bar = 200 µm. (H and I) Pulmonary metastases of canine iUC. Note the uneven labeling for FR. Scale bar = 75 µm. All images were collected with an Eclipse E400 microscope equipped with a Plan Fluor 40×/0.75 and a Nikon Plan Fluor 100×/1.30 oil objectives. Images were acquired with a DS-Fi2 camera controlled with the Digital Sight DS-L3 controller and the NIS-Elements F (4.60.00 version) software. Figures were prepared using the GIMP (version 2.8.22 version) image processing software.

In the bone marrow core biopsy samples, the membrane and cytoplasm of precursor cells within the myeloid lineage had moderate to marked immunoreactivity to FR (Figure 4). Strong immunoreactivity in both the cytoplasm and membrane was noted in more mature granulocytic cells in the bone marrow cores. Heterogeneous, mild to moderate immunoreactivity was noted in megakaryocytes in all six core biopsy samples. No immunoreactivity was observed in erythroid precursors and lymphoid cells within the bone marrow. In blood smears, FRs were detected in both the membrane and cytoplasm in greater than 85% of granulocytes in 15 out of 16 canine cases (Figure 4). This included moderate immunoreactivity in neutrophils and mild immunoreactivity in eosinophils. Immunoreactivity to FRs was not detected on erythrocytes, platelets, or lymphocytes in blood smears.

### Necropsy findings and FR expression in primary iUC and metastatic sites

Necropsy was performed on 21 of the 28 dogs in the trial. Histopathologically-confirmed metastatic disease was found in 14 dogs (67%) at necropsy. This included four dogs with nodal metastasis, two dogs with distant metastasis, and eight dogs with both nodal and distant metastasis. Sites of distant metastases included lung, liver, spleen, muscle, small intestine, colon, brain, adrenal gland, and skin. Eleven of the 21 dogs undergoing necropsy had received additional therapy after failing EC0531. Heterogeneity in FR expression in regards to the percentage of immunoreactive cells, intensity of immunoreactivity, and location of immunoreactivity (membrane, cytoplasmic) was found within primary tumors, and between primary and metastatic tumors (Figure 4). In 11 dogs, samples were available to compare FR expression between multiple (two to four) sites within the primary tumor from the same dog. In six cases, the FR expression was similar in all areas of the primary tumor sampled within the same dog. In five cases, heterogeneity was noted within the primary tumor, ranging from strong positive (>50% immunoreactive cells, strong intensity of immunoreactivity) to negative FR expression within different areas of the same tumor. Heterogeneity was also noted between metastases from the same case (Figure 4). Positive FR expression was noted in metastatic nodes in three of seven cases and in distant metastases in three of nine cases for which samples were available. There was not a consistent change in IHC findings between pre-treatment biopsies and biopsies collected at necropsy.

## DISCUSSION

The results of this canine trial provide strong support for the evaluation of folate-tubulysin in human clinical trials. Folate-tubulysin was well tolerated in pet dogs and had clinical activity in the setting of an aggressive, spontaneously-arising cancer. The work clearly demonstrates that conjugating tubulysin to folate expands the therapeutic index. Whereas unconjugated tubulysin is too toxic for human trials, conjugation of tubulysin to folate produces a drug with the predicted safety to be tested in a clinical setting [12–14]. The tolerability of EC0531 in dogs compares favorably to that of other cytotoxic agents tested in canine clinical trials [25–27]. Whereas no dogs were removed from the EC0531 study due to toxicity, other studies, such as those with cisplatin, had greater than 50% of dogs withdrawn due to drug toxicity [28]. The results of this trial are even more encouraging considering the majority of the dogs presented with advanced age, cancer stage, and/or co-morbidities.

Conjugation of tubulysin to folate not only created tolerability, it also appears to have restored a good therapeutic window. Peak plasma drug concentrations in dogs receiving 0.28 mg/kg EC0531 were approximately 700 times higher than concentrations required to kill FR-expressing tumor cells *in vitro* [14]. It is noted that the pharmacokinetic assays were performed on samples from dogs receiving 0.28 mg/kg EC0531, as initial findings indicated that the MTD would likely be 0.28 mg/kg. The MTD, however, was ultimately determined to be slightly lower than this at 0.26 mg/kg.

Clinical benefit of EC0531 treatment was appreciable in the form of tumor responses of PR in 11% of dogs, and SD in 60% of dogs, for a clinical benefit rate of 71%. To help put these numbers into context, typical outcomes with standard of care drugs (COXis, chemotherapy, and combinations of chemotherapy and COXis) could be considered. COXi treatment has resulted in PR in 17–20% of dogs, SD in 33–71% of dogs, and clinical benefit in 55–88% of dogs [26–28]. The responses of single agent chemotherapy (PR 0–22%, SD 8–70%, clinical benefit 8–92%), have been improved upon with the addition of a COXi to the treatment protocol [25–28]. For example, in a randomized trial, PR was noted in 22% of dogs receiving vinblastine alone and in 58% of dogs receiving vinblastine plus a COXi [26]. Tumor responses with vinblastine alone included 22% PR, and 70% SD [26]. The median PFI in dogs treated with EC0531 was 105 days (range 24–649 days) for all dogs, 200 days (range 56–296 days) for dogs with SD, and 162, 251, and 649 days, respectively for dogs with PR. To help put these numbers into context, in trials of other anticancer drugs (vinblastine, mitoxantrone, cisplatin, carboplatin, with or without a COXi) in dogs with iUC, the median PFI has ranged from 41 to 199 days [25–28]. It is reasonable to conclude that sustained SD achieved by dogs in this trial was the result, at least in large part, of EC0531 treatment. In addition, it is important to note that the owners of some dogs with stable tumor size reported improvement in quality of life and decreased lower urinary tract signs.

Canine neutrophil and neutrophil precursors were found to express FRs, and this could have contributed to the neutropenia observed in the dogs, although the effect of non-targeted tubulysin is unknown. The observed neutropenia also dictated the dosing interval (every two weeks) as time between doses was required for bone marrow recovery. Human neutrophils, however, do not express FRs, and therefore considerably higher doses and more frequent doses of folate-tubulysin conjugates may be possible in humans.

Heterogeneity of FR expression was neither surprising nor does it diminish enthusiasm for further study of folate-targeted therapy. Heterogeneity of FR expression has previously been observed within primary canine and human iUC tumors and between canine primary and metastatic tumors [7, 24]. It is important to note that targeted agents given to patients with heterogeneous tumors are expected to have value in at least two settings. First, targeted therapies are expected to be key components of combination therapies. Second, targeted agents can have antitumor effects that extend beyond the initial target cells, due to “bystander effects”. This can include activation of apoptotic death in cells being targeted and the release of toxic metabolites to inhibit neighboring cells, as well as other mechanisms [29]. Apoptotic cell death also creates immunologic effects. In one example, in a xenogeneic mouse model study, tubulysin-treated cancer cells were found to prevent tumor formation at distant sites through the activation of cytotoxic T-cells and enhanced memory response [30]. In addition, EC0531 is like many other cancer drugs in that antitumor effects are observed with two-week dosing intervals, but with a short drug half-life (< 30 minutes). This indicates mechanisms other than direct rapid cell kill are likely to be involved. “Off-target” immunologic effects are currently generating intense interest as these could lead to antitumor effects that go beyond the direct anti-proliferative mechanisms of cancer drugs [30–31].

In this study, it was not possible to know if neutrophil depletion could have had any role, positive or negative, in the antitumor activity of the EC0531. In humans with iUC, an elevated neutrophil-to-lymphocyte ratio in peripheral blood or neutrophil infiltration in the tumor is thought to be an indicator of deleterious systemic inflammation and has been associated with a poor prognosis [32]. Neutrophils can produce pro-tumorigenic cytokines and pro-angiogenic vascular endothelial growth factor [33, 34]. However, neutrophils can also exert antitumor effects especially when present as N1 type neutrophils [35]. Although the duration of the neutropenia in dogs in this study was of short duration, an effect on the antitumor effects of EC0531 could not be ruled out. In contrast to the reported worse prognosis in humans with higher peripheral blood neutrophil-to-lymphocyte ratios, the neutrophil-to-lymphocyte ratio at the start of EC0531 treatment in dogs in this study was not associated with treatment outcome.

In conclusion, the safety and antitumor activity of EC0531 observed in this aggressive cancer in dogs provides compelling support for human clinical trials of folate-tubulysin. The naturally-occurring pet dog model of iUC incorporates the same clinical issues that affect cancer treatment in humans including tumor heterogeneity, aggressive cancer behavior, multiple drug resistance mechanisms, and limited tolerance for drug toxicity. Thus, folate-tubulysin should be considered especially well vetted for drug safety and antitumor activity, and an appropriate choice for human trials in cancers that overexpress FRs.

## MATERIALS AND METHODS

### Study overview

The work included a phase I/II clinical trial and pharmacokinetic analysis of the folate-tubulysin conjugate, EC0531 (Endocyte Inc., West Lafayette, IN, USA), in dogs with naturally-occurring iUC. Dose escalation and determination of the MTD was followed by expansion of a cohort of dogs treated at the MTD to further assess antitumor activity. Following the observation of myelosuppression in dogs receiving EC0531, an assessment of FR expression in canine white blood cells (WBCs) and WBC precursors was also performed. The canine clinical trial was conducted at the Purdue University Veterinary Teaching Hospital (PUVTH) following the guidelines and approval of the Purdue Animal Care and Use Committee and the Clinical Review Board. The study was open to dogs presenting to the PUVTH between October 2013 and July 2015. During that time, the owners of dogs with iUC were informed of the treatment options including this trial, and provided detailed information about the trial. Written consent by the dog owner was required for the dog to participate in the trial.

### Subject eligibility

Inclusion criteria consisted of dogs with histologically-diagnosed lower urinary tract iUC in which FRs were expressed in >50% of tumor cells (detected by methods described below), expected survival time of at least six weeks, and serum creatinine <2.0 mg/dL (normal reference range 0.5 to 1.5 mg/dL). The expected survival time was based on the health status of the dog as determined by medical history, physical exam, CBC (complete blood cell count), serum biochemical profile, urinalysis, and imaging (thoracic/abdominal radiography, abdominal ultrasonography). Dogs treated with prior therapies were eligible for the trial if cancer progression was noted in response to prior therapy or if treatment had been discontinued for a minimum of four weeks prior to receiving EC0531. Concurrent use of a COXi and EC0531 was permitted if either cancer progression had occurred despite COXi prior to enrollment, or if COXi therapy was necessary for relief of pain from arthritis or other comorbidities.

### Treatment and trial design

EC0531 was given as an intravenous (IV) bolus every two weeks at a starting dose of 0.2 mg/kg. The starting dose and frequency were based on data from laboratory dogs and from a pilot study in pet dogs with iUC in which a neutrophil nadir was observed four to seven days after treatment (*n* = 10 dogs). The 10 pet dogs in the pilot study included six neutered male dogs and four spayed female dogs of a variety of breeds and weighing between 5 and 46 kg. Twice weekly dosing was initially instituted, but was not continued due to dose limiting-neutropenia and anorexia. The weekly dose interval was also suspended due to neutropenia.

Dogs in dosage cohorts were enrolled using a 3+3 trial design. Inter-patient and intra-patient dose escalation was permitted. Drug tolerability was assessed by owner questionnaire, physical exam, CBC the day of treatment and seven days following treatment, and biochemistry panel at four-week intervals. Toxicity was graded using VCOG-CTCAE criteria [36]. The EC0531 dose was increased by 0.02 mg/kg in each subsequent dose cohort if no dose limiting toxicity (DLT) was recorded. DLT was defined as an adverse event (AE) > grade 2. When a grade 3 AE occurred, the cohort was expanded to at least six dogs including six new dogs, i.e. six dogs that had not received prior EC0531 at lower doses. If more than one grade 3 AE or any grade 4 or 5 AE occurred, then six dogs were enrolled at the previous cohort. The MTD was defined as the highest dose tolerated by six dogs with no more than one grade 3 AE and no grade 4–5 AE. Intra-patient dose escalation was permitted if no AEs had occurred after two doses of the drug had been administered.

In individual dogs, the EC0531 dose was reduced by 10% if grade 2 toxicity was noted, and by 20% if grade 3 or higher toxicity was noted. Treatment was delayed by one week if the neutrophil count was <3,000/mm^3^ or platelet count <100,000/mm^3^ the day treatment was due. Dogs that experienced cancer progression or unacceptable toxicity following dose adjustments were eligible to receive other therapies off study.

In order to estimate antitumor activity, the cohort of dogs receiving the MTD was expanded with the goal to assess antitumor effects in a minimum of 24 dogs treated at or above the MTD [37]. The effects of EC0531 on the tumor were assessed by cystosonography using a specific standardized protocol [38] and physical exam including rectal exam at four-week intervals. Complete cancer staging (thoracic radiography with left lateral, right lateral, and ventrodorsal projections; abdominal radiography with right lateral and ventrodorsal projections; and complete abdominal ultrasonography) was performed at eight-week intervals. The response in the primary tumor was determined by comparing volume measurements by cystosonography as follows: complete remission (CR, no cancer detected), partial remission (PR, ≥50% decrease in tumor volume and no new tumor lesions), stable disease (SD, <50% change in tumor volume and no new tumor lesions), and progressive disease (PD, ≥50% increase in tumor volume or the development of new tumor lesions) [26]. Metastatic lesion response was assessed by RECIST criteria [39]. Progression free interval (PFI) was defined as the time from start of EC0531 treatment until PD of local or metastatic lesion(s). Permission to perform a necropsy was requested at the time of the dog’s death (death from cancer or other causes). Tissues collected at necropsy were examined histologically and immunohistochemically to detect FR expression in primary and metastatic lesions.

### Folate receptor assessment

Tumor FR expression was assessed by either immunohistochemistry (IHC) of iUC cells from primary tumor tissues collected by cystoscopic biopsy, or immunocytochemistry (ICC) of iUC cells from urine sediment collected in the previous four weeks of trial enrollment. Folate uptake in distant metastases was assessed by nuclear scintigraphy as previously described [24]. Immunohistochemistry to detect FRs in tissues was performed as previously described [24]. An ICC protocol was established in order to detect FRs in iUC cells in urine. Slides for ICC were fixed in cold acetone, followed by hydration, and then formalin-fixation. Antigen retrieval and detection with a rabbit polyclonal anti-FR antibody (PU17, Endocyte, Inc., West Lafayette, IN, USA) were performed. Paired negative control slides were run with Universal Negative Control Serum (Biocare Medical, Concord, CA, USA). Immunoreactivity was detected by immunoperoxidase-DAB with hematoxylin counterstain. The results obtained using urine smears immunostained with PU17 were compared with immunohistochemically detected FR expression on paired tumor samples from the same dog (*n* = 10). Immunocytochemistry and IHC were also used for detection of FR expression in neutrophils and neutrophil precursors. Immunocytochemistry was performed on peripheral blood smears from 16 dogs, and IHC was performed on bone marrow core biopsies from six dogs from histological archives. These samples were from dogs that had neither neutropenia nor neoplasia in their bone marrow.

### Pharmacokinetic analyses

Plasma pharmacokinetic analysis was conducted in six dogs sequentially enrolled in the trial. Blood was collected at 0, 2, 10, and 30 minutes, and at 1, 2, 8, and 24 hours after treatment.

Tubes were centrifuged, and plasma collected and stored at –80° C. In exploratory analysis, three of these dogs also had cystoscopy-guided collection of tumor tissue 3 hours after treatment, which was also stored at –80° C until analysis.

For the analysis of EC0531 concentrations in plasma, the EC0531 was extracted from 100 µL of dog plasma by 96-well solid phase extraction (SPE). Internal standard was added to all samples prior to extraction using a Strata X-A SPE plate (Phenomenex, Torrance, CA, USA). The eluate was collected, evaporated, and finally reconstituted with 85/15 water/acetonitrile with 40 mM mannitol. The extracted samples were injected into a UPLC/MS/MS system (Waters, Milford, MA, USA) using a BEH C18 reverse phase column (Waters) implementing a mobile phase gradient. Incurred sample concentrations were then back-calculated against a calibration curve prepared in blank dog plasma.

EC0347 (parent tubulysin compound) and other potential metabolites were also assessed in plasma samples. The EC0347 was extracted from 100 µL of dog plasma by 96-well supported liquid extraction (SLE). Internal standard was added to all samples prior to extraction using a 200 µL SLE+ plate (Biotage, Charlotte, NC, USA). The eluate was collected, evaporated, and reconstituted with acetone prior to sealing and heating the collection plate for 1 hour. After incubation, the extracts were again evaporated and finally reconstituted prior to injection. The extracted samples were injected into a UPLC/MS/MS system (Waters) using a BEH shield RP18 reverse phase column (Waters) and eluted under isocratic conditions. EC0347 was detected by monitoring the hydrazone form of the compound following derivatization with acetone. Incurred sample concentrations were then back-calculated against a calibration curve prepared in blank dog plasma. For the analysis of EC0531 and EC0347 in iUC tissue samples, the samples were homogenized with four volumes of 3% mannitol in 50 mM citrate buffer containing N-maleoyl-ß-alanine and acetic acid. Samples were homogenized in buffer with a bead beater, or hand held homogenizer. Aliquots (100 µL) of each sample were spiked with 400 µL internal standards in acetone. The extracts were sealed and incubated at 55° C for 1 hour. The extracts were then evaporated to remove acetone, and reconstituted. Finally, reconstituted samples were analyzed by injection into a UPLC-MS/MS system (Waters) to evaluate concentrations of EC0531 and metabolites against a prepared calibration curve. The parent compound, EC0347, would be expected to be released in tissues.

### Statistical analysis

Stepwise Cox regression analysis was performed to assess the relationship between predictor variables and progression free survival (PFI). The following variables were assessed: age, weight, sex, peripheral blood neutrophil-to-lymphocyte ratio at the start of treatment, tumor location (bladder vs urethra/prostate vs both), treatment beginning at < MTD or ≥ MTD, pretreatment with chemotherapy, concurrent use of COXi, IHC/ICC score, and best tumor response (PD vs SD vs PR/CR). Variables with *p*-values < 0.25 in univariate models were then included in multivariate modeling. Variable(s) with a *p*-value < 0.05 in the final multi-variable Cox regression model were considered to have a significant effect on PFI.

## Abbreviations

iUC: invasive urothelial carcinoma
FR: folate receptor
MTD: maximum tolerated dose
PK: pharmacokinetic
DLT: dose limiting toxicity
PR: partial remission
PD: progressive disease
SD: stable disease
PFI: progressive free interval
IHC: immunohistochemistry
G: grade
